# Incidence and predictors of iron deficiency anaemia in parturients undergoing elective caesarean section at a tertiary hospital in New Zealand: a retrospective, observational cohort study

**DOI:** 10.1186/s12884-021-04121-9

**Published:** 2021-09-22

**Authors:** Pablo L. de Vena Franks, Andrew Y. Pan, Manpreet K. Gill, Angela M. K. Cross, Katy L. Konrad, Nicholas J. Lightfoot

**Affiliations:** 1 Department of Anaesthesia and Pain Medicine, Counties Manukau Health, Auckland, 2025 New Zealand; 2 Department of Obstetrics and Gynaecology, Counties Manukau Health, Auckland, 2025 New Zealand; 3 Department of Anaesthesiology, University of Auckland, Auckland, 1142 New Zealand

**Keywords:** Elective caesarean section, iron deficiency anaemia, Pregnancy outcomes

## Abstract

**Background:**

Worldwide, iron deficiency anaemia in pregnancy is a significant problem which can be especially problematic when delivery is by caesarean section, a procedure associated with significant blood loss. Optimising iron stores pre-delivery remains an overarching goal. We aim to measure the incidence of iron deficiency anaemia in patients undergoing elective caesarean section at our institution and determine any associated predictors, as well as adverse outcomes.

**Methods:**

A retrospective, observational cohort study of patients presenting for elective caesarean section over a two-year period. Patient data was collected from hospital electronic records. Iron deficiency anaemia was defined a haemoglobin < 110 g/L and a ferritin < 30 μg/L in the three-month period prior to delivery. The primary aim was to establish the incidence of iron deficiency anaemia at the time of delivery and any associated predictors. Secondary outcomes included any association between the primary outcome and complications defined by the hospital discharge complication coding system, as well as an evaluation of the number of blood tests carried out antenatally per trimester.

**Results:**

One thousand and ninety-three women underwent caesarean section over the study period and 16.2% had iron deficiency anaemia. Patients with iron deficiency anaemia were more likely to be of Māori and Pacific Island ethnicity, have a greater booking body mass index, be younger and have a greater parity. Pre-operative anaemia was associated with a greater likelihood of post-operative blood transfusion.

**Conclusions:**

There remains potential for optimisation of iron deficiency anaemia in our local population undergoing elective caesarean section.

**Supplementary Information:**

The online version contains supplementary material available at 10.1186/s12884-021-04121-9.

## Background

Pregnancy-related iron deficiency (ID) and iron deficiency anaemia (IDA) remain a significant problem worldwide [1]. IDA can lead to significant morbidity for both the parturient and her baby including increased rates of post-partum infection, blood transfusion and post-natal depression, as well as preterm birth and lower neonatal birth weights [2, 3]. Iron is essential for many physiological processes and requirements increase during high metabolic states, such as pregnancy. As such, patients with reduced iron stores prior to pregnancy can progress to frank IDA as their gestation increases.

More than 20 million women worldwide undergo caesarean section (CS) annually, and the procedure can be associated with significant blood loss [4]. Therefore, determining the impact of pre-existing IDA is particularly important. Optimisation of iron stores is increasingly recognised as one way of reducing risk in surgical patients, both by correcting IDA and by avoiding allogenic blood transfusion. A haemoglobin (HB) level of < 110 g/L is still the most established cut-off for defining anaemia in pregnancy, although this has been challenged, given that higher HB targets are used in the non-obstetric population, commonly 120 g/L [5, 6]. In order to optimise HB levels in the pregnant population it is important that practitioners recognise the burden of IDA and specific patient demographics in their community.

The primary aim of this study is to establish the incidence of IDA in South Auckland in patients undergoing elective CS and to determine the predictors which are associated with IDA. Secondary aims include any associated adverse outcomes following CS in those with IDA compared to those without. In addition, we will evaluate the number of blood tests carried out per trimester to audit our own practice and assess whether we are optimally identifying women with IDA. We will also identify the proportion of patients who received iron therapy pre-operatively to optimise iron stores. Oral iron is not routinely recommended at our institution unless there is evidence of iron deficiency and its management follows local guidelines, which are further elucidated in Additional file 1.

We believe our findings provide a recent point of comparison as to the incidence, predictors and implications of IDA in women undergoing elective CS. This information is useful for other centres, not only for the presented incidence of IDA and ID, but also for the data surrounding the number of women receiving the appropriate blood tests in the antenatal period. The population of South Auckland is unique with the large numbers of Māori and Pacific peoples who may have considerable medical comorbidities in addition to metabolic demands of pregnancy. This may provide an interesting insight for those reading this paper in other countries.

## Methods

The STROBE checklist was used to structure this study [7]. An out of scope exemption was granted by the New Zealand Health and Disability Ethics Committee. Approval was obtained from Counties Manukau Health and the hospital’s Woman’s Health Research Committee. Counties Manukau Health serves a population of more than 500,000 residents. The region is ethnically diverse and is home to the second largest Māori population and the largest population of Pacific Islanders with almost 40% of the populace born outside of New Zealand [8]. Each year in our institution approximately 6500 parturients deliver with 27% of women undergoing CS, of which close to one third are elective.

This is a retrospective, observational cohort study of patients presenting for elective CS at Counties Manukau Health in Auckland, New Zealand, between the 1st January 2017 and 31st December 2018. This study period was chosen in order to provide a sufficiently large sample size for analysis and recent data that would be relevant to our current and evolving practice. To help minimise selection bias, the inclusion criteria was any patient delivering by elective CS during these dates. The study assessed only elective, rather than emergency, CS patients in an attempt to focus on those patients most likely to benefit from targeted pre-operative iron therapy. Patients were identified through their National Health Index (NHI) number using the Patient Information Management System (PiMS). Demographic, laboratory and outcomes data were collated from the hospitals electronic data warehouse, managed by the HealthAlliance. Attempts were made to address absent data by running additional searches through ancillary databases. These were limited to our own institution as we were unable to access results from external facilities. By focussing the study primarily on clearly defined laboratory and demographic data we hoped to minimise information bias, however, outcomes and iron therapy data could remain a potential source of information bias as they are reliant upon accurate documentation.

The specific variables and blood test results which were extracted are detailed in Additional file 2. Complications were identified using discharge ICD-10 codes and a review of medical records. These were defined a priori and are in agreement with the standards suggested by the COMPAC-StEP group [9]. The derivation of the ‘Obstetric Infection’ composite outcome is shown in Additional file 3.

Data are presented for the overall cohort then stratified by patients with valid HB and ferritin recordings. To correct for factors which were associated with anaemia on univariate comparisons in the overall cohort, propensity score matching was undertaken first with a logistic regression model then matching using a tolerance of 0.005 between the anaemic and non-anaemic groups. Analyses as per the overall cohort were repeated to assess the impact of anaemia on the primary and secondary outcomes once the identified confounders were corrected.

As per the current World Health Organisation (WHO) definition of anaemia in pregnancy, anaemia was defined as a HB < 110 g/L while ‘no anaemia’ was a HB ≥110 g/L. IDA was defined as a HB recording < 110 g/L and a ferritin < 30 μg/L within the three-month period prior to CS, while ID was defined as a ferritin recording within the same period of < 30 μg/L. Non-Anaemic Iron Deficiency (NAID) was defined as those with a ferritin < 30 μg/L and a HB ≥110 g/L and ‘normal’ was defined as having both a HB and ferritin ≥110 g/L and ≥ 30 μg/L. In those with multiple observations, the recording closest to surgery was used. Given the preponderance of iron deficiency anaemia, those with anaemia without iron deficiency were excluded.

The primary outcome was the incidence of IDA in patients undergoing elective CS. Secondary outcomes consisted of associations between IDA, demographic variables and complications. The comparison groups were the ‘normal’ and NAID cohorts, respectively. For completeness, statistical tests were completed between each of the three groups and *p*-values presented as appropriate. Prior to study conduct, a formal power calculation was not completed given the primary outcome was an estimate of the prevalence of iron deficiency anaemia in our population.

The number of blood tests for HB and iron studies by trimester were accessed and a comparison made between those with anaemia and no anaemia. This comparison was made as HB recordings were more frequent than ferritin levels and most protocols for iron therapy in pregnancy rely on a reduced HB level due to the prevalence of ID in pregnancy [10]. The incidence of anaemia was defined using the minimum reported HB value in those with more than one recording. Blood tests for the third trimester were defined as between 27 weeks’ gestation and 2 weeks prior to the CS date to allow time for iron therapy to be effective, if implemented. Trimesters were defined as the definitions of the National Institute of Child Health and Human Development [11].

Statistical analyses were completed in SPSS Version 26.0. Results are presented as number (percentage), median (interquartile range) as appropriate. Comparisons were made with the Fisher Exact test (two by two contingency tables) or the Chi square test with a Yates correction for categorical variables and the Mann Whitney U-test (between two outcomes) or the Kruskal-Wallis test (more than two outcomes) for continuous variables, respectively. Non-parametric statistical tests were used to maximise statistical power due to non-normal distribution of parameters. To allow for multiple statistical tests, a Šidák correction was applied to counteract the effect of multiple comparisons. The critical *p*-value is presented in the post-script of each of the tables, with significant *p*-values highlighted in bold.

## Results

Between the 1st January 2017 and 31st December 2018, 1093 women underwent elective CS. Of these, 933 (85.3%) had a HB recording within the 3 months before CS and 532 (48.7%) had both a HB and ferritin recording, see Table 1. In the overall cohort, 18.0% patients were anaemic. Of those anaemic patients, 48.7% also had a ferritin recording.

**Table 1:** Demographics Overall Cohort

	Overall Cohort	Anaemia HB < 110 g/L	No Anaemia HB ≥110 g/L	p-value
Number	1093 (100.0%)	168 (18.0%)	765 (82.0%)	–
Age (years)	32.0 (28.2–35.5)	30.2 (26.1–34.8)	32.3 (28.8–35.8)	**< 0.001**
Weight (kilograms)	79.0 (64.0–98.6)	90.6 (72.0–107.7)	76.0 (63.0–97.0)	**< 0.001**
Body Mass Index (kg/m^2^)	29.5 (24.7–36.3)	33.0 (27.2–39.4)	28.7 (24.1–35-6)	**< 0.001**
Body Mass Index Classification				**< 0.001**
- Underweight	40 (3.7%)	1 (0.6%)	32 (4.2%)	
- Normal	255 (23.3%)	22 (13.1%)	197 (25.8%)	
- Overweight	275 (25.2%)	41 (24.4%)	194 (25.4%)	
- Obese	523 (47.8%)	104 (61.9%)	342 (44.7%)	
Ethnicity				**< 0.001**
- African	15 (1.4%)	1 (0.6%)	11 (1.4%)	
- Chinese	31 (2.8%)	1 (0.6%)	23 (3.0%)	
- Indian	184 (16.8%)	14 (8.3%)	150 (19.6%)	
- Middle Eastern	26 (2.4%)	8 (4.8%)	17 (2.2%)	
- NZ European / Pakeha	242 (22.2%)	24 (14.3%)	171 (22.4%)	
- NZ Maori	127 (11.6%)	32 (19.0%)	74 (9.7%)	
- Other	2 (0.2%)	0 (0.0%)	1 (0.1%)	
- Other Asian	54 (4.9%)	5 (3.0%)	43 (5.6%)	
- Other European	68 (6.2%)	4 (2.4%)	53 (6.9%)	
- Pacific Islander	301 (27.6%)	75 (44.6%)	185 (24.2%)	
- South East Asian	42 (3.8%)	4 (2.4%)	36 (4.7%)	
Prior Pregnancies				
- Gravida	3 (2–4)	3 (2–5)	3 (2–4)	**< 0.001**
- Para	1 (1–2)	2 (1–3)	1 (1–2)	**< 0.001**
Number Prior Caesarean Sections	1 (0–1)	1 (0–2)	1 (0–1)	0.008
Plurality				0.10
- Singleton	1054 (96.4%)	158 (94.0%)	738 (96.5%)	
- Twins	37 (3.4%)	9 (5.4%)	26 (3.4%)	
- Triplets or above	2 (0.2%)	1 (0.6%)	1 (0.1^)	
Gestation at Delivery (weeks)	39.0 (38.3–39.3)	39.0 (38.1–39.3)	39.0 (38.3–39.3)	0.65
Singleton Foetal Weight (grams)	3500 (3170–3890)	3660 (3330–4010)	3460 (3140–3850)	**< 0.001**
Preoperative Blood Test Results				
- Haemoglobin Obtained	933 (85.4%)	168 (100.0%)	765 (100.0%)	–
- Ferritin Obtained	532 (48.7%)	103 (61.3%)	429 (56.1%)	–
- Haemoglobin and Ferritin Obtained	532 (48.7%)	103 (61.3%)	429 (56.1%)	–
- Haemoglobin (g/L)	121 (113–128)	104 (100–107)	123 (118–129)	**< 0.001**
- Haemoglobin < 120 g/L	424 (38.8%)	168 (100.0%)	256 (33.5%)	**< 0.001**
- Ferritin (mcg/L)	19 (11–32)	13 (7–21)	21 (13–34)	**< 0.001**
- Ferritin < 30mcg/L	266 (49.9%)	83 (80.6%)	287 (66.9%)	**< 0.001**

Demographic data for the overall cohort of 1093 patients with comparison made between those with anaemia (HB < 110 g/L) and those with no anaemia (HB ≥110 g/L)

Data presented as number (percent) or median (interquartile range) as appropriate

Sidak correction for multiple tests – revised p-value for statistical significance becomes 0.0034 – significant results highlighted in bold

Following adjustment for multiple tests, several differences were found between those with and without anaemia in the overall cohort. These are shown in Table 1. Propensity score matching using patient age, maternal ethnicity, Body Mass Index (BMI), maternal gravida and parity, plurality, gestation at birth and number of prior CS at a match tolerance of 0.005 yielded a match between 138 (83.6%) of anaemic to non-anaemic parturients, see Table 2. There were no differences in the baseline characteristics between the two groups. Those with anaemia had a lower HB following CS (*P* < 0.001) and were more likely to receive an allogenic blood transfusion (*P* < 0.001). Despite their lower post-surgical HB, the change in HB between the closest paired pre-operative and post-operative measurement was lower in the anaemic group (5 (− 2–11) versus 8 (1–14) g/L, *P* = 0.008), yet did not meet the corrected threshold for significance. There was no difference in critical care utilisation (*P* = 1.00), hospital length of stay (*P* = 0.77) or infectious complications (*P* = 0.60) between the propensity score matched groups.

**Table 2:** Propensity Score Match

	Anaemia HB < 110 g/L	No Anaemia HB ≥110 g/L	p-value
Number	138 (50.0%)	138 (50.0%)	–
Age (years)	30.7 (26.7–35.2)	31.2 (27.9–34.2)	0.82
Weight (kilograms)	90.0 (72.0–107.0)	83.5 (66.0–110.0)	0.46
Body Mass Index (kg/m^2^)	32.8 (27.1–38.9)	31.7 (25.8–40.7)	0.47
Body Mass Index Classification			0.36
- Underweight	1 (0.7%)	5 (3.6%)	
- Normal	19 (13.8%)	20 (14.5%)	
- Overweight	34 (24.6%)	37 (26.8%)	
- Obese	84 (60.9%)	76 (55.1%)	
Ethnicity			0.95
- African	1 (0.7%)	1 (0.7%)	
- Chinese	1 (0.7%)	1 (0.7%)	
- Indian	12 (8.7%)	17 (12.3%)	
- Middle Eastern	5 (3.6%)	9 (6.5%)	
- NZ European / Pakeha	21 (15.2%)	17 (12.3%)	
- NZ Maori	21 (15.2%)	21 (15.2%)	
- Other Asian	5 (3.6%)	7 (5.1%)	
- Other European	4 (2.9%)	3 (2.2%)	
- Pacific Islander	64 (46.4%)	57 (41.3%)	
- South East Asian	4 (2.9%)	5 (3.6%)	
Prior Pregnancies			
- Gravida	3 (2–5)	3 (2–5)	0.58
- Para	2 (1–3)	1 (1–3)	0.25
Number Prior Caesarean Sections	1 (0–2)	1 (0–2)	0.80
Plurality			0.52
- Singleton	132 (95.7%)	129 (93.5%)	
- Twins	6 (4.3%)	8 (5.8%)	
- Triplets or above	0 (0.0%)	1 (0.7%)	
Gestation at Delivery (weeks)	39.0 (38.1–39.3)	39.0 (38.1–39.1)	0.81
Singleton Foetal Weight (grams)	3690 (3328–4010)	3520 (3175–4010)	0.20
Preoperative Blood Test Results			
- Haemoglobin (g/L)	104 (100–107)	119 (115–127)	**< 0.001**
- Haemoglobin < 120 g/L	138 (100.0%)	72 (52.2%)	**< 0.001**
- Ferritin Result Obtained	85 (61.6%)	97 (70.3%)	0.16
- Ferritin (mcg/L)	11 (7–20)	19 (11–35)	**< 0.001**
- Ferritin < 30mcg/L	70 (82.4%)	65 (67.0%)	0.03
Iron Therapy Pre-Operatively			
- No Iron	59 (42.8%)	46 (33.3%)	0.14
- Oral Iron	71 (51.4%)	90 (65.2%)	0.007
- Intravenous Iron	22 (15.9%)	3 (2.2%)	**< 0.001**
Length of Stay (hours)	55.6 (52.1–77.7)	56.2 (35.4–79.3)	0.77
Postoperative Blood Test Results			
- Haemoglobin (g/L)	97 (89–107)	112 (105–120)	**< 0.001**
- Haemoglobin Measured	135 (97.8%)	135 (97.8%)	1.00
- Change (Pre to Postoperative, g/L)	5 (−2–11)	8 (1–14)	0.008
- Anaemia Postoperatively (110 g/L)	117 (86.7%)	59 (43.7%)	**< 0.001**
- Haemoglobin < 120 g/L	133 (98.5%)	99 (73.3%)	**< 0.001**
Blood Transfusion	13 (9.4%)	1 (0.7%)	**0.001**
Critical Care Post-Partum			
- Intensive Care Unit	1 (0.7%)	0 (0.0%)	1.00
- High Dependency Unit	0 (0.0%)	0 (0.0%)	–
- Either / Both	1 (0.7%)	0 (0.0%)	1.00
Infectious Complications Related to Pregnancy	9 (6.5%)	6 (4.3%)	0.60

Propensity score matching between the anaemic and non-anaemic groups. Matching using patient age, maternal ethnicity, Body Mass Index (BMI), maternal gravida and parity, plurality, gestation at birth and number of prior CS at a match tolerance of 0.005 yielded a match between 138 (83.6%) of anaemic to non-anaemic parturients

Data presented as number (percent) or median (interquartile range) as appropriate

Sidak correction for multiple tests – revised p-value for statistical significance becomes 0.0018 – significant results highlighted in bold

In those with a valid HB and ferritin recording, and after excluding those with anaemia without ID, the incidence of IDA was 16.2%. The incidence of NAID was 56.1 and 27.7% had neither ID nor anaemia. Overall, 80.5% (83/103) of parturients with anaemia had IDA. The median overall preoperative HB was 121 g/L (interquartile range 113–128) and median ferritin was 19 (11–31)μg/L, see Table 3.

**Table 3:** Demographics of Patients with Valid Haemoglobin and Ferritin Recordings

	Overall Cohort with Valid HB and Ferritin	IDA HB < 110 g/L Ferritin < 30mcg/L	NAID HB ≥110 g/L Ferritin < 30mcg/L	Normal HB ≥110 g/L Ferritin ≥30mcg/L	p-values
Number	512 (100.0%)	83 (16.2%)	287 (56.1%)	142 (27.7%)	–
Age (years)	32.3 (28.1–35.8)	29.8 (26.0–33.8)	32.4 (28.6–36.1)	32.6 (29.1–35.6)	**0.002**^a^ **0.001**^b^ 1.00 ^c^
Weight (kilograms)	76.1 (63.5–100.0)	87.2 (73.0–102.5)	76.0 (64.0–102.2)	72.3 (61.5–93.0)	0.009 ^a^ 0.19 ^b^ 0.24 ^c^
Body Mass Index (kg/m^2^)	28.9 (24.9–36.6)	32.9 (27.1–37-6)	28.7 (24.3–36.7)	27.2 (24.3–33.5)	**0.001**^a^ 0.008 ^b^ 0.70 ^c^
Body Mass Index Classification					
- Underweight	26 (5.1%)	1 (1.2%)	16 (5.6%)	9 (6.3%)	**0.002**^a^
- Normal	110 (21.5%)	9 (10.8%)	66 (23.0%)	35 (24.6%)	0.006 ^b^
- Overweight	138 (27.0%)	20 (24.1%)	77 (26.8%)	41 (28.9%)	0.85 ^c^
- Obese	238 (46.5%)	53 (63.9%)	128 (44.6%)	57 (40.1%)	
Ethnicity					
- African	3 (0.6%)	0 (0.0%)	1 (0.3%)	2 (1.4%)	**0.001**^a^
- Chinese	16 (3.1%)	0 (0.0%)	7 (2.4%)	9 (6.3%)	0.009 ^b^
- Indian	92 (18.0%)	11 (13.3%)	51 (17.8%)	30 (21.1%)	0.09 ^c^
- Middle Eastern	15 (2.9%)	5 (6.0%)	6 (2.1%)	4 (2.8%)	
- NZ European / Pakeha	98 (19.1%)	10 (12.0%)	65 (22.6%)	23 (16.2%)	
- NZ Maori	63 (12.3%)	16 (19.3%)	35 (12.2%)	12 (8.5%)	
- Other	1 (0.2%)	0 (0.0%)	1 (0.3%)	0 (0.0%)	
- Other Asian	24 (4.7%)	3 (3.6%)	14 (4.9%)	7 (4.9%)	
- Other European	27 (5.3%)	1 (1.2%)	19 (6.6%)	7 (4.9%)	
- Pacific Islander	147 (28.7%)	36 (43.4%)	77 (26.8%)	34 (23.9%)	
- South East Asian	26 (5.1%)	1 (1.2%)	11 (3.8%)	14 (9.9%)	
Prior Pregnancies					
- Gravida	3 (2–4)	3 (2–5)	3 (2–4)	2 (2–3)	0.007 ^a^ 0.91 ^b^ 0.01 ^c^
- Parity	1 (1–2)	2 (1–3)	1 (1–2)	1 (1–2)	**0.001**^a^ 0.42 ^b^ 0.009 ^c^
Number Prior Caesarean Sections	1 (0–2)	1 (0–2)	1 (0–2)	1 (0–1)	0.08 ^d^
Plurality					
- Singleton	485 (94.7%)	78 (94.0%)	273 (95.1%)	134 (94.4%)	1.00 ^a^
- Twins	26 (5.1%)	5 (6.0%)	1 (0.3%)	8 (5.6%)	0.74 ^b^
- Triplets or above	1 (0.2%)	0 (0.0%)	13 (4.5%)	0 (0.0%)	0.69 ^c^
Gestation at Delivery (weeks)	38.5 (38.1–39.3)	39.0 (38.1–39.4)	39.0 (38.1–39.3)	38.8 (38.0–39.1)	0.06 ^d^
Singleton Foetal Weight (grams)	3470 (3130–3860)	3640 (3190–3960)	3510 (3140–3930)	3330 (3040–3690)	**0.002**^a^ 0.46 ^b^ 0.01 ^c^
Preoperative Blood Test Results					
- Haemoglobin Result Obtained	512 (100.0%)	83 (100.0%)	287 (100.0%)	142 (100.0%)	–
- Haemoglobin (g/L)	121 (113–128)	104 (101–107)	122 (116–129)	125 (118–131)	**< 0.001**^a^ **< 0.001**^b^ 0.16 ^c^
- Haemoglobin < 120 g/L	241 (47.1%)	83 (100.0%)	115 (40.1%)	43 (30.3%)	**< 0.001**^a^ **< 0.001**^b^ 0.06 ^c^
- Ferritin Result Obtained	512 (100.0%)	83 (100.0%)	287 (100.0%)	142 (100.0%)	–
- Ferritin (mcg/L)	19 (11–31)	10 (7–16)	15 (10–21)	41 (34–63)	**< 0.001**^a^ **0.001**^b^ **< 0.001**^c^
- Ferritin < 30mcg/L	370 (72.2%)	83 (100.0%)	287 (100.0%)	0 (0.0%)	–

This table shows the demographic data for all those with both a valid HB and ferritin recording, 512 in total. The incidence of IDA amongst this group was 16.2%. The demographic parameters on the left are compared between the overall cohort and those with IDA, NAID and normal blood results

Abbreviations – IDA – iron deficiency anaemia; NAID – non anaemic iron deficiency

Data presented as number (percent) or median (interquartile range) as appropriate

Sidak correction for multiple tests – revised p-value for statistical significance becomes 0.0037 – significant results highlighted in bold

^a^ iron deficiency anaemia versus normal ^b^ iron deficiency anaemia versus non anaemic iron deficiency ^c^ non anaemic iron deficiency versus normal

^d^ no significant difference – multiple testing not completed

Significant demographic differences were found between the IDA and those with normal HB and ferritin recordings, specifically, patient age (*P* = 0.002), body mass index (*P* = 0.001), ethnicity (*P* = 0.001), parity (*P* = 0.001) and singleton foetal weight (*P* = 0.002). See Additional file 4 for additional graphs relating to gravida and parity data. Those with IDA had a significantly lower baseline HB than either the normal or NAID groups (*P* < 0.001 for both comparisons). The ferritin levels were significantly different between the three groups with the lowest levels seen in those with IDA, higher levels in those with NAID and the greatest levels in the normal patients, which reflects the definitions of the respective conditions.

Post-operatively, those with IDA were more likely to be anaemic when compared to both the normal and NAID groups (*P* < 0.001 for both), see Table 4. The median post-operative HB was 99 (89–108)g/L in the IDA group vs 118 (109–127) in the normal group. The IDA group were also significantly more likely to receive a blood transfusion. There was no difference found in the rate of critical care utilisation or the number of infectious complications related to pregnancy between the three groups.

**Table 4:** Outcomes of Patients with Valid Haemoglobin and Ferritin Recordings

	Overall Cohort	IDA HB < 110 g/L Ferritin < 30mcg/L	NAID HB ≥110 g /L Ferritin <30mcg/L	Normal HB ≥110 g/L Ferritin ≥30mcg/L	p-values
Length of Stay (hours)	55.1 (34.4–75.8)	55.4 (52.1–74.3)	54.9 (33.3–76.0)	55.3 (35.1–75.8)	0.27
Postoperative Blood Test Results					
- Haemoglobin Measured	506 (98.8%)	81 (97.5%)	285 (99.3%)	140 (98.6%)	–
- Haemoglobin (g/L)	113 (104–122)	99 (89–108)	113 (105–122)	118 (109–127)	**< 0.001**^a^ **< 0.001**^b^ **0.004**^c^
- Change (Pre to Postoperative, g/L)	8 (0–15)	4 (− 3–12)	10 (3–16)	7 (− 1–14)	0.27 ^a^ < 0.001 ^b^ 0.02 ^c^
- Haemoglobin < 110 g/L Postoperatively	212 (41.9%)	66 (81.5%)	104 (36.5%)	42 (30.0%)	**< 0.001**^**a**^ **< 0.001**^b^ 0.19 ^c^
- Haemoglobin < 120 g/L Postoperatively	361 (71.3%)	79 (97.5%)	201 (70.5%)	81 (57.8%)	**< 0.001**^a^ **< 0.001**^b^ 0.01 ^c^
Blood Transfusion	10 (2.0%)	7 (8.4%)	3 (1.0%)	0 (0.0%)	**0.001**^a^ **0.002**^b^ 0.55 ^c^
Critical Care Post-Partum					
- Intensive Care Unit	0 (0.0%)	0 (0.0%)	0 (0.0%)	0 (0.0%)	–
- High Dependency Unit	2 (0.4%)	0 (0.0%)	2 (0.7%)	0 (0.0%)	- ^a^ 1.00 ^b^ 1.00 ^c^
- Either / Both	2 (0.4%)	0 (0.0%)	2 (0.7%)	0 (0.0%)	- ^a^ 1.00 ^b^ 1.00 ^c^
Infectious Complications Related to Pregnancy	27 (5.3%)	7 (8.4%)	10 (3.5%)	10 (7.0%)	0.80 ^a^ 0.07 ^b^ 0.14 ^c^

Outcomes are compared here between those with valid HB and ferritin results. As with Table 3, comparison made between the overall cohort and those with IDA, NAID and with normal blood results. The median post-op HB was 99 in the IDA group vs 118 in the normal group. The IDA group were significantly more likely to receive a blood transfusion. No difference detected in the rate of critical care utilisation or number of infectious complications related to pregnancy between the three groups

Data presented as number (percent) or median (interquartile range) as appropriate

Sidak correction for multiple tests – revised *p*-value for statistical significance becomes 0.0057– significant results highlighted in bold

^a^ iron deficiency anaemia versus normal ^b^ iron deficiency anaemia versus non anaemic iron deficiency ^c^ non anaemic iron deficiency versus normal

^d^ no significant difference – multiple testing not completed

When parturient blood results were summated by trimester of pregnancy as defined in the methods, 27.6, 29.2 and 50.9% of patients in the first, second and third trimester, respectively, had at least one valid HB level identified, see Table 5. For ferritin, 18.9, 20.5 and 40.1% of parturients had valid measurements in the corresponding trimesters. There was no difference between the proportion of anaemic and non-anaemic parturients with valid HB or ferritin measurements at all time periods. The incidence of anaemia by trimester using a patient’s minimum recorded HB level in those with valid recordings was 7.3% (22/302, 1st trimester), 30.4% (97/319, 2nd trimester) and 34.2% (190/556, 3rd trimester).

**Table 5:** Iron Parameters

	Overall Cohort	Anaemia HB < 110 g/L	No Anaemia HB ≥110 g/L	p-value
Haemoglobin Recording Obtained				
- 1st Trimester	302 (27.6%)	46 (27.4%)	217 (28.4%)	0.85
- 2nd Trimester	319 (29.2%)	59 (35.1%)	240 (31.4%)	0.36
- 3rd Trimester	556 (50.9%)	103 (61.3%)	453 (59.2%)	0.66
Ferritin Recording Obtained				
- 1st Trimester	207 (18.9%)	30 (17.9%)	157 (20.5%)	0.46
- 2nd Trimester	224 (20.5%)	40 (23.8%)	177 (23.1%)	0.84
- 3rd Trimester	438 (40.1%)	74 (44.0%)	364 (47.6%)	0.44
Anaemia – < 110 g/L				
- 1st Trimester	22 (7.3%)	11 (23.9%)	11 (5.1%)	–
- 2nd Trimester	97 (30.4%)	35 (59.3%)	57 (23.8%)	–
- 3rd Trimester	190 (34.2%)	95 (92.2%)	95 (21.0%)	–
Iron Therapy Pre-Operatively				
- No Iron	426 (39.0%)	69 (41.1%)	289 (37.8%)	0.43
- Oral Iron	630 (57.6%)	84 (50.0%)	456 (59.6%)	0.03
- Intravenous Iron	64 (5.9%)	26 (15.5%)	34 (4.4%)	**< 0.001**
Thresholds for IV Iron Therapy				
- HB < 110 g/L and Ferritin < 20 mcg/L	71 (6.5%)	71 (42.3%)	0 (0.0%)	–
- HB < 110 g/L and Ferritin <30 mcg/L	83 (7.6%)	83 (49.4%)	0 (0.0%)	–
- HB < 120 g/L and Ferritin < 20 mcg/L	158 (14.5%)	71 (42.3%)	87 (11.4%)	–
- HB < 120 g/L and Ferritin <30 mcg/L	198 (18.1%)	83 (49.4%)	115 (15.0%)	–

This table shows the number of HB and ferritin recordings obtained for the overall cohort of patients in each trimester. Comparison columns between the number of blood tests obtained in the anaemic and non-anaemic groups. The number of patients who had documented oral and iv iron therapy in each group is shown in the fourth row. The final row shows the effect of using different thresholds for IV iron therapy based on the HB and ferritin levels recorded. For example, if a more liberal threshold of HB < 120 g/L and Ferritin <30 mcg/L were to be used, a greater proportion (49.4%) of the anaemic cohort would receive IV iron when compared to a stricter threshold of HB < 110 g/L and Ferritin < 20 mcg/L.

3rd trimester data presented from 27 weeks until 2 weeks prior to delivery to allow time for any potential intervention to have an effect

Data presented as number (percent) or median (interquartile range) as appropriate

Sidak correction for multiple tests – revised p-value for statistical significance becomes 0.017 – significant results highlighted in bold

Similar numbers of patients received iron preparations between the anaemic and non-anaemic groups (*P* = 0.43). Those without anaemia at term were more likely to receive oral iron (*P* = 0.03), while those in the anaemic group received intravenous iron more frequently (*P* < 0.001). In the overall cohort, the utilisation of intravenous iron was 5.9%. Using differing standards to qualify for iron therapy led to increasing numbers of patients reaching theses thresholds in both the anaemic and non-anaemic groups, see Table 5.

## Discussion

To our knowledge, this is the first study from New Zealand which presents a complete data set in patients undergoing elective CS including the incidence of IDA, the complications associated with IDA and the rate of blood testing during the antenatal period. Most of data which exists, such as that from the WHO, focuses on women who are pregnant and does not stratify by the mode of delivery or by parturient ethnicity. Given the significant morbidity associated with complications following CS, the information presented provides some insight as to the effect of anaemia on parturients.

We have shown that the incidence of IDA in patients undergoing elective CS at our institution is 16.2%, which is in keeping with pre-existing data for New Zealand [12]. NAID was also common at 56.1%. The median preoperative ferritin level was 19 μg/L and 10 μg/L in the IDA group. There were several predictors of IDA including those with an increased booking BMI, younger parturients, who had delivered more children previously and those of New Zealand Māori or Pacific Island ethnicity. Patients who had IDA preoperatively were significantly more likely to be anaemic postoperatively and received more allogenic blood transfusions.

Following propensity score matching, the IDA and ‘normal’ cohorts were balanced with regards to patient age, gravida and parity, ethnicity and number of prior deliveries. Those with IDA pre-delivery remained more likely to be anaemic following delivery and received blood transfusions more frequently. Interestingly, our study found an association between women with IDA and greater singleton foetal weight, which is contrary to the existing literature that highlights maternal anaemia as a key risk factor for lower birth weight [13]. It is likely that the contradictory association found in this study is a result of confounders. The anaemia and IDA cohort in this study had a greater proportion number of parturients of Māori and Pacifica ethnicities, which are populations associated with greater rates of diabetes and obesity [14]. This, in turn, could explain the higher birth weights observed as a result of pregnancy-induced diabetes.

The crude incidence of anaemia from all causes increased as the antenatal period progresses, from 7.3% in the first trimester to 34.2% in the third trimester. These values are greater than those reported at term as many patients had their HB measured on multiple occasions, perhaps to monitor the effect of oral iron therapy. The median HB at the time of delivery was 121 g/L. As it stands, in the 3rd trimester up to 2 weeks prior to surgery, approximately 50% of patients had a valid HB level and 40% a valid ferritin, an interval which is likely the minimum window for intravenous iron supplementation to be effective. These results are indicative of a patient population where there is a significant opportunity to optimise iron stores and HB prior to delivery. Although these data looked at all cause anaemia, we have shown that upwards of 80% is explained by ID in our cohort. Attempts were made to identify alternative causes of anaemia, such as vitamin deficiency, chronic inflammatory disease or thyroid problems, however, there were limited laboratory data obtained during pregnancy to conclusively identify these states. On review of discharge ICD-10 coding, these diagnoses were infrequently observed meaning that for approximately 20% of patients, no cause for their anaemia was identified. Alternative risk factors for anaemia, such as diet were not included in the study data set and would be difficult to assess accurately using the study methodology. Explanations such as recent blood donation or post-traumatic / post-surgical anaemia would also seem less likely.

It is well established that ID and IDA in pregnancy is associated with sequelae to both the mother and child [15–18]. Those with IDA were exposed to transfusion more frequently, which can lead to increased rates of Rhesus alloimmunisation, impaired healing and sepsis [19]. Although we were unable to demonstrate this finding, perhaps due to the low rates of transfusion and infectious complications, the strong relationship between anaemia and transfusion should be at the forefront of practitioners minds when considering treatment for mild to moderate anaemia in both obstetric and non-obstetric populations.

Iron plays an important role in cognition, development and emotional states, and the adverse effects of IDA may be subtle [20–22]. In multiple groups, associations exist between ID and depression [23, 24]. One randomised study has shown that early iron supplementation in pregnant women improves iron reserves and significantly reduced rates of post-partum depression [23]. In addition to low iron stores, a HB level of less than 120 g/L is a recognised predictor for developing post-partum depression [25].

Although the WHO recommends supplemental daily dose elemental iron for all pregnant women in all settings, this recommendation is not routinely followed in New Zealand. Our institution follows a local protocol that aims to treat ID or IDA if there is evidence of these states on antenatal blood tests. Our guidelines recommend maintenance dose iron if there is evidence of ID but no anaemia, and treatment dose iron in the presence of IDA or if the ferritin is < 20 μg/L, with a view to achieving an HB level > 110 g/L prior to delivery. The full details of our local protocol can be found in Additional file 1. This approach to ‘treat when indicated’, rather than to supplement all women, is supported by a recent international consensus review in which the recommendation in areas of low prevalence, such as New Zealand, is to check ferritin levels in non-anaemic women and offer iron supplementation based on these results [6].

Our institution uses ferrous fumurate as the oral iron of choice, and although oral iron therapy was prescribed and dispensed frequently, the use of intravenous iron (ferric carboxymaltose), remains infrequent. At our hospital, to use intravenous iron, a 2nd or 3rd trimester HB of less than 100 g/L with a ferritin of < 20 μg/L is required. Our data suggest that in patients who are anaemic immediately prior to elective CS, the process has often been apparent since at least the second trimester, if not before. Given the potential consequences of low iron stores it could be argued that targeting a higher ferritin level would prompt earlier treatment with oral iron, leading to higher HB levels at term and is unlikely to lead to harm.

Modern intravenous iron preparations have the advantage of enhanced tolerability [26]. Following administration, ferritin levels peak within 10 days and the rise in HB concentration is seen within 14–21 days [27, 28]. This is ideal in circumstances where there is limited time between the detection of anaemia and CS. Intravenous iron is also effective at increasing HB levels post-partum when compared to oral iron as it circumvents the effects of hepcidin in preventing oral iron absorption [29].

To make optimal use of iron, it is vital to have contemporaneous blood tests. In our population the first step would encompass more regular testing across trimesters starting early in the antenatal period with clear referral pathways for treatment. It is our opinion that the current WHO guideline which drives our protocol for iron supplementation is too restrictive with regards both the ferritin and HB thresholds [29]. There is also considerable debate as to the ferritin level which should be targeted to ensure normal maternal and foetal development. This can be made more complex in patients with chronic disease states where the iron studies can be difficult to interpret [30–32]. However, it is likely beneficial for patients if a ferritin level > 30 μg/L was targeted antenatally.

This study has several limitations. We were unable to match blood test results for a small proportion of women who underwent elective CS. This may relate to parturients from outside our hospital catchment or those where laboratory testing was completed by external laboratories. The data associated with iron administration also has limitations. Patients may receive a prescription for iron, and have it dispensed, but there is no way to ensure the mediation was consumed as directed. As this is a retrospective series, its duration is also a limitation. By studying a two-year period, we were able to obtain a reasonable sample size and a meaningful estimate of the incidence and predictors of IDA. Increasing the study period could potentially increase the precision of these estimates. The downside would be the time required to extract the additional data and could also make the data less current. As such, we feel that the time period studied represents a balance between relevance of results and statistical accuracy. We also elected to study parturients undergoing elective CS rather than a cohort undergoing both elective and emergency CS. CS is accompanied by moderate blood loss and by limiting our sample to elective procedures we were able to assess the impact of anaemia in a more predictable patient group. Although we were able to identify statistically significant predictors of IDA in our population, we should bear in mind that these might not be modifiable or causal. We believe that our findings may be generalisable to other populations undergoing the same operation in other countries.

## Conclusion

We have identified a significant proportion of patients with both IDA and ID in women undergoing elective CS. This is associated with increased rates of postoperative anaemia and exposure to allogenic blood. To further improve outcomes at our institution, enhanced preoperative screening for IDA and ID across all trimesters is required which may lead to increased utilisation of iron therapy. We have already begun to use these results as an education tool for all staff caring for women who may require elective CS and have drafted a new obstetric anaemia protocol to improve the delivery if iron therapy.

## Supplementary Information

**Additional file 1.**

**Additional file 2.**

**Additional file 3.**

**Additional file 4.**

## Data Availability

The vast majority of data generated and analysed for this study are included in this published article. Any datasets not used in this article are available from the corresponding author on reasonable request.

## Abbreviations

ID: iron deficiency
IDA: iron deficiency anaemia
CS: caesarean section
HB: haemoglobin
NHI: National Health Index
PiMS: Patient Information Management System
WHO: World Health Organisation
NAID: Non-Anaemic Iron Deficiency
BMI: Body Mass Index (BMI),
