# Supplementary material for: Incidence and predictors of iron deficiency anaemia in parturients undergoing elective caesarean section at a tertiary hospital in New Zealand: a retrospective, observational cohort study
Source: BMC Pregnancy Childbirth. 2021 Sep 22;21:645. doi: 10.1186/s12884-021-04121-9 (PMC8459509; doi:10.1186/s12884-021-04121-9)
Supplement: Supplementary file 1 — Additional file 1. [file 12884_2021_4121_MOESM1_ESM.pdf]

Guideline: Iron deficiency anaemia in pregnancy and post-partum – prevention and management.

Background / Overview

This guideline describes how to identify, prevent and manage iron deficiency anaemia (IDA) in pregnancy

- Iron deficiency (ID) is the commonest cause of anaemia in pregnancy and there is a higher prevalence of both iron deficiency and iron deficiency anaemia (IDA) in Maaori, Pacific and Indian women. Physiological anaemia of pregnancy must be distinguished from IDA
- Anaemia is defined by the British Committee for Standards in Pregnancy as; Hb <110g/L in the first trimester, Hb<105g/L in the second and third trimester and Hb <100g/L in the post partum period.
- Serum ferritin accurately reflects iron stores in the absence of inflammatory change. It is the first laboratory test to become abnormal as iron stores decrease. A serum ferritin concentration below 15ug/L indicates iron depletion in all stages of pregnancy.
- Treatment should be considered when serum ferritin levels fall below 30ug/L as this indicates early iron depletion which will worsen unless treated.
- A recent audit of women delivering at Middlemore Hospital found that, of women tested, approximately one third had anaemia (Hb <110g/L) or were iron deficient (serum ferritin <12ug/L as per previous guideline) with their first antenatal bloods. Only 37% of these women were treated appropriately.
- Anaemia and iron deficiency anaemia (IDA) can impact on pregnancy and pregnancy outcome. IDA can contribute to maternal morbidity through effects on immune function with increased susceptibility or severity of infections, poor work capacity and performance. Anaemia increases the morbidity and potentially mortality from postpartum haemorrhage and low maternal ferritin is associated with low cord blood ferritin.
- The fetus is relatively protected from the effects of iron deficiency by upregulation of placental iron transport proteins but evidence suggests that maternal iron depletion increases the risk of iron deficiency in the first 3 months of life. Impaired psychomotor and/or mental development are well described in infants with IDA. Iron deficiency prevalence in children under 2 years of age in Auckland is 14%, double the rate in Sydney, Europe and USA.
- Dietary advice should be provided for all pregnant women. Methods of screening, treatment and ferritin/Hb levels which justify treatment are not proven by randomised trials. Therefore the screening methods thresholds recommended for treatment and the regimens advised are decided by consensus of opinion of clinicians rather than based on randomised trials.

Purpose

The aims of this guideline are to reduce the incidence of maternal iron deficiency anaemia at the onset of labour and to improve maternal and fetal/neonatal iron stores in pregnancy and post-partum.

**Document ID:**	A25392	**Version:**	2.0
**Department:**	Obstetric and Gynaecology	**Last Updated:**	29/09/2015
**Document Owner:**	Clinical Leader - Obstetrics	**Next Review Date:**	29/09/2018
**Approved by:**	Maternity Quality Forum	**Date First Issued:**	23/08/2011
**Counties Manukau District Health Board**			

Roles and Responsibilities

This guideline is applicable to medical and midwifery staff responsible for the care for pregnant women who plan to give birth in CMDHB Maternity Facilities.

Guideline

Diagnosis of anaemia and IDA.

- **Clinical symptoms and signs** of IDA in pregnancy are usually non-specific unless there is severe anaemia. Fatigue is the most common symptom. Women may complain of pallor, weakness, headache, palpitations, dizziness, dyspnoea and irritability. IDA may impair temperature regulation and cause pregnant women to feel colder than usual.

- Storage iron is depleted before a fall in Hb and as iron is an essential element in all cells, symptoms of iron deficiency may occur even without anaemia.

Laboratory tests

- Full blood count (FBC), blood film, red cell indices

- A FBC is taken routinely at pregnancy booking. It may show a low Hb, mean cell volume (MCV), mean cell haemoglobin (MCH) and mean cell haemoglobin concentration (MCHC). The blood film may confirm the presence of microcytic hypochromic red cells and characteristic 'pencil cells'. These results are all indicative of IDA.
- Microcytic (Low MCV) and hypochromic (low MCH) indices can also occur in haemoglobionopathies and these should be screened for if the ferritin is not reduced.
- Serum ferritin
- Serum ferritin is a stable glycoprotein which accurately reflects iron stores in the absence of inflammation. It is the first laboratory test to become abnormal as iron stores decrease and it is not affected by recent iron ingestion.
- A serum ferritin concentration <15ug/L indicates iron depletion at all stages of pregnancy.
- There are a variety of levels for treatment quoted but, in general treatment should be considered when serum ferritin levels fall below 30ug/L as this indicates early iron depletion which will worsen unless treated.

A variety of more specialised investigations can be requested from the laboratory but these should not be done routinely, rather after consultation with an Obstetric Physician or Haematologist.

Prevention and Treatment of ID and IDA.

a. Diet

- Physiological iron requirements are three times higher in pregnancy than in menstruating women, with increasing demands as pregnancy advances.
- Dietary advice should be given to all women. In the Growing Up in NZ study only 21% of women adhered to the Ministry of Health guideline for diet in pregnancy which advises 2 or more serves of lean meat, meat alternatives or eggs per day.
- The amount of iron absorption depends upon the amount of iron in the diet, its bioavailability and physiological requirements.
- Haem iron in lean red meat and to a lesser extent fish and chicken is better absorbed (2-3X) and promotes absorption of non-haem iron.
- Non-haem iron is contained in whole grain breads and fortified cereals, vegetables and dried fruit.
- Vitamin C (ascorbic acid) containing foods such as vegetables and fruit as well as fruit juice increases non-haem iron absorption.
- Some cereals, legumes and nuts as well as chapattis contain phytates which inhibit absorption of non-haem iron. Oxalates in spinach and rhubarb similarly reduce absorption of non-haem iron.
- Tannins in tea and coffee inhibit iron absorption when consumed with a meal or shortly after.
- Calcium reduces absorption of both haem and non-haem iron.
- The Ministry of Health has a free pamphlet 'Eating for Healthy Pregnant Women' available on line and for ordering.

b. Oral iron

- Once women become iron deficient in pregnancy it is not possible to ensure repletion through diet alone and oral supplementation is required.
- Oral iron is an effective, cheap and safe way to replace iron. The available formulas show only marginal differences between one another in efficiency of absorption.
- The recommended dose of **elemental iron** for treatment of iron deficiency is 100-200mg daily. Higher doses should not be given as absorption is saturated and side effects increase.
- **Women should be counselled as to how to take oral iron supplements correctly. This should be on an empty stomach, 1 hour before meals, with a source of vitamin C such as orange juice to maximise absorption. Other medications or antacids should not be taken at the same time.** If side effects occur, iron supplements may be taken with food to improve tolerability.

Preparations currently available

Iron Salt	Brand Name	Iron salt content per tablet	Elemental iron content per tablet	Community Subsidy	Dose
**Ferrous fumarate**	Ferro-Tab tablets	200mg	65mg	Fully subsidised	**Maintenance:** 200mg PO daily **Treatment:** 200mg PO BD or TDS **Supply:** 3 months
	Ferro-F-Tabs tablets	310mg (plus 350microgram folic acid*)	100mg	Fully subsidised	**Treatment:** 1 tab PO daily or BD **Supply:** 3 months
**Ferrous sulfate**	Ferodan oral liquid	300mg **(per 10mL)**	60mg **(per 10mL)**	Fully subsidised	**Maintenance:** 10mL PO daily **Treatment:**15-30mL PO in three divided doses **Supply:** 2OP or 3 months
**Ferrous sulfate, dried**	Ferrograd controlled release tablets**	325mg	105mg	Fully subsidised	**Treatment:** 325mg PO daily **Supply:** 3 months
	Ferrograd F controlled release tablets**	325mg (plus 350microgram folic acid*)	105mg	Partially subsidised	**Treatment:** 1 tab PO daily **Supply:** 3 months
	Ferrograd C controlled release tablets**	325mg (plus 562.4mg ascorbic acid)	105mg	Not subsidised	**Treatment:** 1 tab PO daily **Supply:** 3 months

*Quantity of folic acid not adequate for prevention of neutral tube defects (≥0.8mg folic acid required)
**Note: poor absorption of controlled release formulation, use second line after ferrous fumarate

c. Intravenous iron

Iron deficient pregnant women who are intolerant of oral iron or who are non-responsive to 4 weeks of oral iron therapy can be offered ferric carboxymaltose (Ferinject) as the first line intravenous iron treatment.

For a full list of Contraindications and Precautions plus the detailed Dosage and Administration information please refer to the Guideline: Intravenous Ferric (Iron) Carboxymaltose (Adults).

<u>Special notes</u>

Ferric carboxymaltose is **contra-indicated in the <u>first</u> trimester of pregnancy**

Pregnancy is listed as a precaution – the manufacturer does not recommend the use of Ferric Carboxymaltose in pregnancy but several studies support the safe and efficacious use of this treatment.

Breastfeeding Transfer of iron from Ferric Carboxymaltose to human milk was negligible (<1%) in clinical studies. This treatment can therefore be used in the post partum setting.

PREVENTION and MANAGEMENT OF ID and IDA

FIRST ANTENATAL VISIT

- **Dietary advice, prescribe iodine and folic acid**
- **Check full blood count and ferritin**

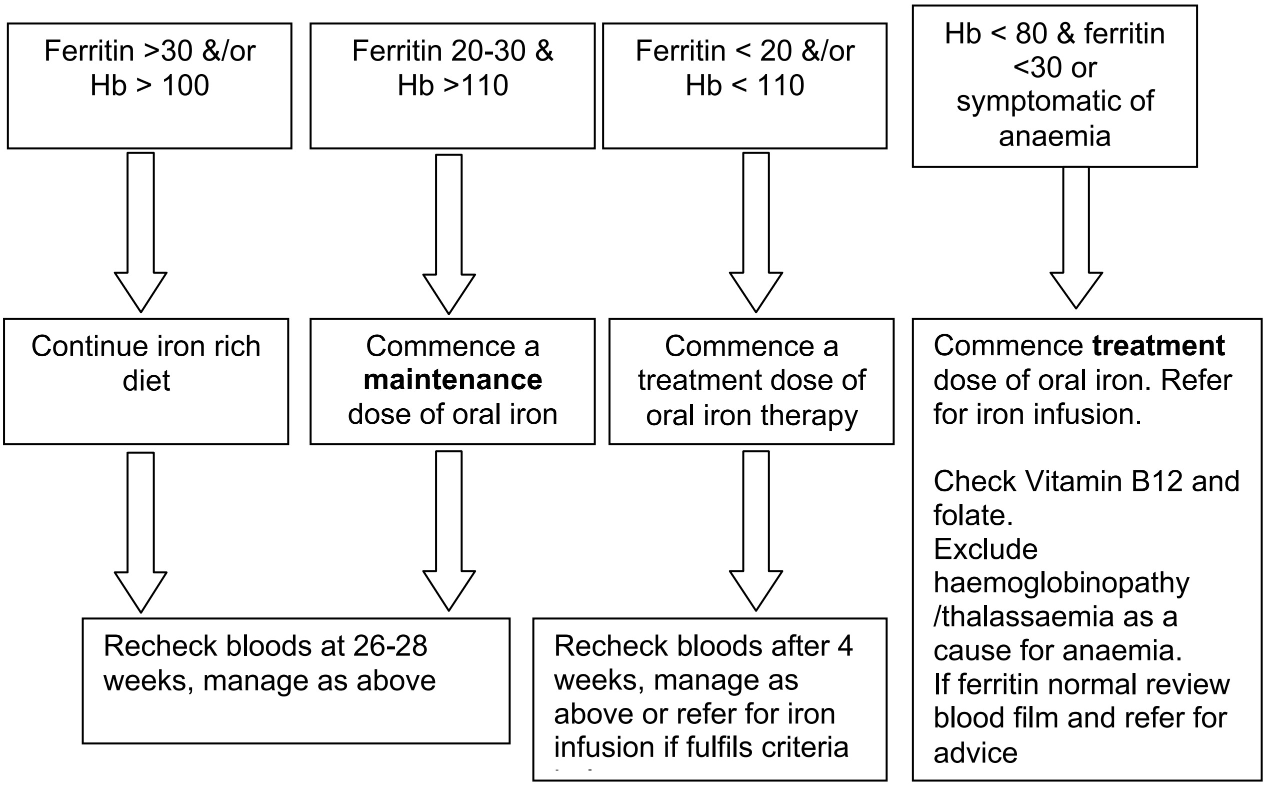

Indications for Iron Infusion (Ferric Carboxymaltose(Ferinject))
ANTENATAL 2nd and 3rd **trimester**
Hb <100g/L and/or ferritin <20ug/L AND one or more of the following

1. Fetal compromise eg IUGR
2. Unresponsive to oral iron after 4 weeks (<10g/L rise in Hb and ferritin remains low)
3. Intolerant to oral iron
4. Severe anaemia (Hb <80g/L)

POSTNATAL
One or more of the following

1. Hb <90g/L
2. Hb <100g/L with symptoms of anaemia
3. Patient has received a blood transfusion (to replenish iron stores)

Process for booking women into B&A for a Ferric Carboxymaltose infusion

- The DAC (Day Assessment Clinic) in B&A runs planned sessions for iron infusions on Monday and Thursday for eligible women over 20/40 pregnant.
- Appointments can be requested via a referral to Maternity Administration for secondary grading. Fax 8055 (internal) 2760055 (external) or via a secondary referral on BadgerNet.
- Referrals **must** include maternal weight at booking, current Hb and ferritin (within a week) and allergy status, so that the dose of Ferric Carboxymaltose can be prescribed and prepared prior to the appointment
- It is the responsibility of the referrer to counsel the woman about the iron infusion, including location and timing (approximately 1 hour), and the indication for the infusion. See page 9 for possible adverse effects.
- There are no contraindications to driving following an iron infusion but women may have one adult accompanying them in DAU for the infusion.
- Women will be phoned to arrange a planned appointment
- The DAC is run by Registered Nurses; they will not be doing antenatal checks.
- Appointments will be for one hour, commencing every half hour.

Prescribing and administration of Ferric Carboxymaltose

- Doses have been calculated by Pharmacy using the Ganzoni formula and put into table form for ease of prescribing (see below)
- The Ganzoni formula:

Booking weight (kg) x (Target Hb - current Hb [g/L]) x (0.24) + iron reserve

- The target Hb is **110g/L for pregnancy** and **150 g/L for post-partum**
- The iron reserve is 15mg/kg (weight of 35kg or below) or **500mg** (weight above 35kg)
- For women over 90kg use weight of 90kg
- **Prescribe on the 'Once Only' page as 'Ferric Carboxymaltose (dose) in 100ml of 0.9% normal saline to run over 15 minutes'.**
- **Doses from 500mg to 1000mg can be given in a volume of 100mL-250mL normal saline if 100mL bags are not available.**
- **Undiluted Ferric Carboxymaltose is not recommended in pregnancy as the published data in pregnancy is only in the infusion form.**
- A **maximum single dose of 1000mg** of Ferric Carboxymaltose can be given in a single infusion. If weight is 35kg or under the dose should not exceed 20mg iron/kg. When the maximum dose is >1000mg, the additional iron (second dose) should be given at least one week after the first dose as per the table.

Concurrent Blood transfusion

- For a woman receiving a red blood cell (RBC) transfusion the iron content of the packed RBCs needs to be taken into consideration when calculating the dose of iron. Each unit of packed RBCs with a volume of 300mL contains approximately 200mg of iron in the form of haemoglobin haeme.

Antenatal Dose Calculation for Ferinject Infusion

Hb (g/L)	80		83		85		88	90	93	95	98	100	105
**Weight (Kg)**	**1st Dose**	**2nd Dose**	**1st Dose**	**2nd Dose**	**1st Dose**	**2nd Dose**	**ONE dose only**	**ONE dose only**	**ONE dose only**	**ONE dose only**	**ONE dose only**	**ONE dose only**	**ONE dose only**
**50**	800		800		800		700	700	700	600	600	600	500
**55**	900		800		800		700	700	700	700	600	600	500
**60**	900		800		800		800	700	700	700	600	600	500
**65**	900		900		900		900	800	800	700	700	600	600
**70**	1000		1000		1000		900	900	800	800	700	700	600
**75**	1000	100	1000		1000		900	900	800	800	800	700	600
**80**	1000	100	1000	100	1000		1000	900	900	800	800	700	600
**90**	1000	200	1000	100	1000	100	1000	1000	900	900	800	800	600

Note: For weight of 65Kg or less, dose is rounded down to nearest 100mg

For weight of 65Kg or more, dose is rounded up to nearest 100mg

If weight and/or Hb fall outside the table parameters please use Ganzoni formula to calculate dose and follow the above rounding principals

Post-Partum Dose Calculation for Ferinject Infusion

- This can be calculated by the Simplified Method shown below

**Total body weight (kg)**	Total cumulative dose of iron for haemoglobin(Hb) level	
	**Hb less than 100g/L**	**Hb equal to or greater than 100g/L**
35 to 69	1500mg	1000mg
70 or more	2000mg	1500mg

- A **maximum of 1000mg** can be given at any one time (maximum 20mg/kg for patients <35kg) and a second dose should not be given within a week.

Monitoring for Ferinject Infusion

- **Parenterally administered iron preparations can cause hypersensitivity reactions including anaphylactoid reactions, which may be fatal. Therefore, facilities for cardio-pulmonary resuscitation must be available.**

- Record baseline blood pressure (BP), temperature, heart rate (HR) and respiratory rate (RR).
- Enquire about fetal movements and listen for FHR.
- Nurse is to remain with patient during the first 5 minutes after commencement and also record observations at the end of the infusion and 30 minutes after completion of the infusion.
- Blood test to assess for iron and haemoglobin levels should be performed at least one week after last dose of IV iron.

NB: Stop infusion/injection and inform medical staff if any signs of anaphylaxis occur (swelling (including of the neck or mouth), rash, dizziness, difficulty breathing, hypotension).

For a full list of information on administration, stability and adverse effects please refer to the Medsafe data sheet on ferric carboxymaltose or the Intravenous Ferric Carboxymaltose (Adults) Guideline

Adverse Effects associated with Ferinject infusion

NB: Patients must be informed about adverse reactions prior to commencement of administration

Common
- Headache and Dizziness
- Nausea
- Infusion site reaction

Other Adverse Effects
- Hypertension
- Abdominal pain
- Constipation
- Diarrhoea
- Hypophosphataemia
- Hypersensitivity including anaphylactoid reactions (uncommon)
- Angiodema
- Bronchospams
- Anxiety

Management of infusion related reactions

Infusion-related side effects are listed above. If these occur then the infusion should be stopped. If the symptoms persist, stop the infusion and consult the B&A North registrar. A corticosteroid and an antihistamine may be administered if appropriate. If the symptoms resolve, the infusion can be recommenced.

Premedication with corticosteroids and antihistamines may be used for subsequent infusions where patients have had a previous adverse reaction to an iron infusion.

Special considerations

Oral therapy should not commence until at least one week after the last iron injection, as absorption of oral iron is reduced if given with parenteral iron.

Additional information for use in pregnancy

There are two published studies of the use of ferric carboxymaltose antenatally (Froessler et al, BMC, 2014 & Christoph et al, 2012) that include a total of 168 women who received ferric carboxymaltose. One mild hypotensive episode was reported and there were no anaphylactic or anaphylactoid reactions. There are a further three publications (Breymann et al, 2008; Van Wyck et al, 2007 and Seid et al, 2008) in which ferric carboxymaltose was given to a total of 544 women postpartum. There were no reported hypotensive or anaphyactoid reactions, nor other serious adverse events.

d. Intramuscular iron

Prior to the approval of Ferinject as an intravenous infusion, women resistant to or unable to tolerate oral iron were offered intramuscular (IM) iron.

The improved safety profile of Ferinject and the ability to administer it in the primary units means that it should be offered in preference to intramuscular iron.

The use of IM iron should be reserved for women for whom there is a specific contraindication to the use of Ferinject.

Iron polymaltose (Ferrum H) IM injection 50 mg/ml – 2ml ampoules is fully funded. The total dose advised can be calculated from the data sheet and administered as 2 mL by intramuscular injection every second day until the total dose is attained or 4 mL every four days until the total dose is attained.

The commonly stated side effect of skin staining can be reduced if not eliminated by ventrogluteal Z-track injection as recommended in the data sheet. Iron intramuscular injection can be administered in antenatal clinics as well as in general practice. There is a small risk of anaphylaxis with intramuscular iron.

Blood Transfusion

After 38 weeks gestation or one week prior to likely delivery date blood transfusion should be offered only if Hb<70g/L or if symptomatic anaemia and Hb<80g/L.

Intravenous iron can be administered before or after a blood transfusion remembering that one unit of RBCs with a volume of 300mL contains approximately 200mg of iron which should be taken into account when calculating the dose (see above)

Management of Anaemia when Admitted in Labour

- If Hb< 100g/L labour and birth is recommended to be at Middlemore Hospital.
- If Hb ≤ 80g/L for IV cannula and cross match at least 2 units of blood and 1 further unit for every 10 g/L less than 80g/L.
- If Hb > 80g/L g/L and < 100g/L for IV cannula and check blood group and antibody screen. If caesarean section required commence cross matching blood at time of decision.

References

Allen LH. Anaemia and iron deficiency: effects on pregnancy outcome *Am J Clin Nutr (2000);71*(suppl): 1280S-4S

Morton SMB, Grant CC, Wall C R, Atatoa Carr PE, Bandara D K, Schmidt JM, Ivory V, Inskip HM, Camargo Jr CA. (2014). **Adherence to nutritional guidelines in pregnancy: Evidence from the Growing Up in New Zealand birth cohort study.** *Public Health Nutrition, FirstView Article*, 1-11. Doi: 10.1017/S1368980014000482

Pavord S et al; UK Guidelines on the mangement of iron deficiency in pregnancy. British committee for Standards in Haematology July 2011

Grant CC, Wall CR, Brewster D, Nicholson R, Whitehall J, Super L and Pitcher L. Policy Statement on iron deficiency anaemia in pre-school-aged children. *J Paediatrics and Child Health* (2007) 43: 513-521

Milman N. Prepartum anaemia: prevention and treatment. *Ann Hematol* (2008) 87: 949-959

Ministry of Health, Wellington. Food and Nutrition Guidelines for Healthy Pregnant and Breastfeeding Women - A Background Paper (April 2006 - revised November 2008)

Healthcare Logistics. Ferinject injection data sheet 2 April 2015.
http://www.medsafe.govt.nz/profs/datasheet/f/ferinjectinj.pdf (accessed 17/08/2015).

Definitions

Terms and abbreviations used in this document are described below:

Term/Abbreviation	Description
IDA	Iron deficiency anaemia
ID	Iron deficiency
B&A	Birthing and Assessment
FHR	Fetal Heart Rate

Associated Documents

Other documents relevant to this guideline are listed below:

**NZ Legislation**	None
**CMDHB Clinical Board Policies**	None
**NZ Standards**	None
**Organisational Procedures or Policies**	**Guideline:**<br>Intravenous Ferric Carboxymaltose (Adults)<br>Iron polymaltose Infusion(Adults)<br>Iron Sucrose (Venofer)<br>Antenatal Shared Care Guidelines
**Other related documents**	None
