# Supplementary material for: Incidence and predictors of iron deficiency anaemia in parturients undergoing elective caesarean section at a tertiary hospital in New Zealand: a retrospective, observational cohort study
Source: BMC Pregnancy Childbirth. 2021 Sep 22;21:645. doi: 10.1186/s12884-021-04121-9 (PMC8459509; doi:10.1186/s12884-021-04121-9)
Supplement: Supplementary file 2 — Additional file 2. [file 12884_2021_4121_MOESM2_ESM.docx]

Additional file 2 – Data extracted for study conduct

Data extracted from the hospital databases:

- Demographic Information
  - National Health Index (NHI) number
  - Maternal date of birth
  - Age at time of elective CS
  - Maternal ethnicity
  - Weight
  - Height
  - Body Mass Index (BMI) (kg/m^2^)
- Obstetric Information
  - Date of elective CS = Foetal Date of Birth
  - Prior pregnancies
    - Gravida
    - Parity
    - Number of Previous CS
    - Prior pregnancy complications
  - Estimate date of delivery
  - Date of pregnancy registration at Middlemore Hospital
  - Current pregnancy complications
  - Foetal gestation at birth
  - Preoperative iron therapy – oral and intravenous
- Outcomes
  - Critical Care Unit Admission
    - Intensive Care Unit (ICU) Admission
    - High Dependency Unit (HDU) Admission
    - Either ICU or HDU Admission
  - Estimated Blood Loss (EBL) at 24 hours post CS
  - Allogenic blood transfusion
  - Obstetric infection – See Supplementary Appendix Two for derivation
- Blood Test Results – Both preoperative and postoperative measurements with value and time of measurement
  - Complete blood count (CBC)
    - Haemoglobin (HB)
      - Proportion with preoperative anaemia (HB <110g/L)
      - Proportion with preoperative HB <120g/L
      - Proportion with postoperative anaemia (HB <110g/L)
      - Proportion with postoperative HB <120g/L
      - Delta haemoglobin – closest preoperative minus postoperative value
    - Haematocrit, mean cell haemoglobin concentration, mean cell volume, red blood cell count, C-reactive protein
  - Iron studies and indices of anaemia
    - Ferritin
      - Proportion with preoperative ferritin recording
      - Proportion with preoperative ferritin <20mcg/L
      - Proportion with preoperative ferritin <30mcg/L
      - Proportion with preoperative iron deficiency anaemia
    - Iron concentration, transferrin, transferrin saturation, B12, folate
  - Liver function tests
    - Albumin, total bilirubin, conjugated bilirubin
  - Renal function and metabolic indices
    - Creatinine, estimated glomerular filtration rate, urea, albumin to creatinine ratio, protein to creatinine ratio, fasting and random glucose, HBA1c
