# Supplementary material for: Incidence and predictors of iron deficiency anaemia in parturients undergoing elective caesarean section at a tertiary hospital in New Zealand: a retrospective, observational cohort study
Source: BMC Pregnancy Childbirth. 2021 Sep 22;21:645. doi: 10.1186/s12884-021-04121-9 (PMC8459509; doi:10.1186/s12884-021-04121-9)
Supplement: Supplementary file 3 — Additional file 3. [file 12884_2021_4121_MOESM3_ESM.docx]

Additional file 3 – Derivation of ‘Obstetric Infection’ composite outcome

The unique ICD-10 complication codes were reviewed by a study author and Specialist Obstetrician (Dr Angela MK Cross) and codes which could be linked with post CS infectious complications were identified.

The codes identified were:

| ICD-10 | Description |
| --- | --- |
| A560 | Chlamydial infection of lower genitourinary tract |
| A568 | Sexually transmitted chlamydial infection of other sites |
| A600 | Herpesviral infection of genitalia and urogenital tract |
| A6305 | Vulval (venereal) warts |
| B009 | Herpesviral infection, unspecified |
| B349 | Viral infection, unspecified |
| B86 | Scabies |
| B956 | Staphylococcus aureus as the cause of diseases classified to other chapters |
| B961 | Klebsiella pneumoniae [K. pneumoniae] as the cause of diseases classified to other chapters |
| B962 | Escherichia coli [E. coli] as the cause of diseases classified to other chapters |
| B964 | Proteus (mirabilis)(morganii) as the cause of diseases classified to other chapters |
| B9688 | Other and unspecified bacterial agents as the cause of diseases classified to other chapters |
| H109 | Conjunctivitis, unspecified |
| J069 | Acute upper respiratory infection, unspecified |
| J09 | Influenza due to certain identified influenza virus |
| J101 | Influenza with other respiratory manifestations, other influenza virus identified |
| J189 | Pneumonia, unspecified |
| J22 | Unspecified acute lower respiratory infection |
| K521 | Toxic gastroenteritis and colitis |
| K610 | Anal abscess |
| L010 | Impetigo [any organism] [any site] |
| L033 | Cellulitis of trunk |
| O230 | Infections of kidney in pregnancy |
| O234 | Unspecified infection of urinary tract in pregnancy |
| O235 | Infections of the genital tract in pregnancy |
| O752 | Pyrexia during labour, not elsewhere classified |
| O753 | Other infection during labour |
| O85 | Puerperal sepsis |
| O860 | Infection of obstetric surgical wound |
| O862 | Urinary tract infection following delivery |
| O864 | Pyrexia of unknown origin following delivery |
| O983 | Other infections with a predominantly sexual mode of transmission complicating pregnancy, childbirth and the puerperium |
| O984 | Viral hepatitis complicating pregnancy, childbirth and the puerperium |
| O988 | Other maternal infectious and parasitic diseases complicating pregnancy, childbirth and the puerperium |
| T827 | Infection and inflammatory reaction due to other cardiac and vascular devices, implants and grafts |
| U739 | Unspecified activity |
