# Supplementary material for: Incidence and predictors of iron deficiency anaemia in parturients undergoing elective caesarean section at a tertiary hospital in New Zealand: a retrospective, observational cohort study
Source: BMC Pregnancy Childbirth. 2021 Sep 22;21:645. doi: 10.1186/s12884-021-04121-9 (PMC8459509; doi:10.1186/s12884-021-04121-9)
Supplement: Supplementary file 4 — Additional file 4. [file 12884_2021_4121_MOESM4_ESM.docx]

**Additional file 4 - Gravida and Parity Data**

1. Tables

|  | Anaemia Classification 110 YN Preop | | | | | | | |
| --- | --- | --- | --- | --- | --- | --- | --- | --- |
|  | N | | | | Y | | | |
|  | Valid N | Median | Percentile 25 | Percentile 75 | Valid N | Median | Percentile 25 | Percentile 75 |
| Gravida | 765 | 3 | 2 | 4 | 168 | 3 | 2 | 5 |
| Parity | 765 | 1 | 1 | 2 | 168 | 2 | 1 | 3 |

1. Gravida


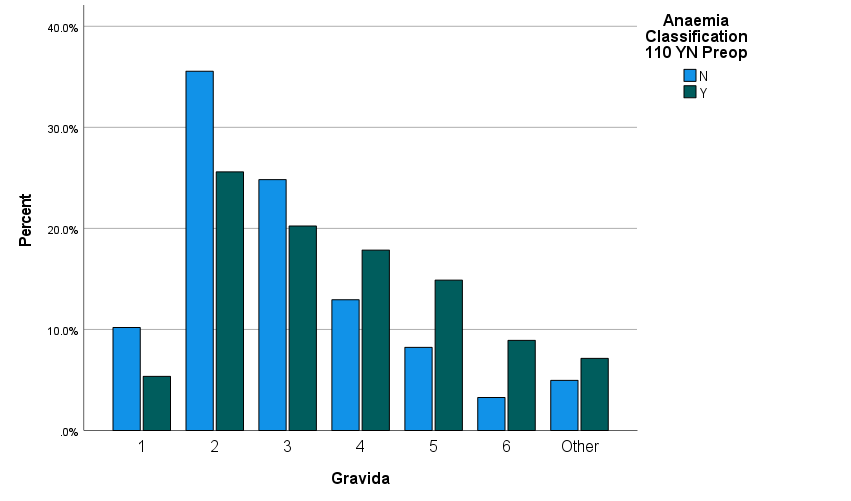


1. Parity


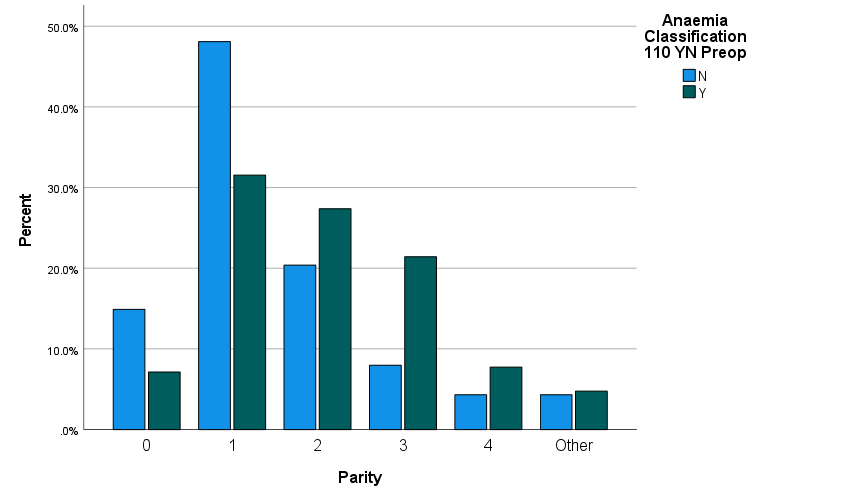


The table and two graphs show the comparative proportions of gravida and parity numbers between the anaemic and non-anaemic groups. The median gravida numbers are equal because the middle 50% of parturients are the same for both groups, but the extremes are higher in the anaemic group due to the disproportionate number of Maori and Pacifica women who have delivered more children.
